# Declining fertility in urban areas

**DOI:** 10.2471/BLT.24.021124

**Published:** 2024-11-01

## Abstract

Declining total fertility rates in urban areas are causing a re-examination of traditional responses to pro-natal policy. Gary Humphreys reports.

Puja Varaprasad wasn’t thinking about having a baby. “My grandmother had eight children and my mother had two. I wasn’t sure I wanted any,” says the forty-year-old shipping lawyer based in Singapore. “It wasn’t something I could talk about openly because childbearing is supposed to be such a gift. In the end I had a boy, and that was enough for me.”

Varaprasad is one of millions of young women worldwide who have opted to have one child or to forego childbearing altogether, a decision which is contributing to a decline in the global total fertility rate (TFR) – the average number of children that a woman would be expected to have over her lifetime.

According to the United Nations Department of Economic and Social Affairs, the global TFR currently stands at approximately 2.25 children per woman, slightly above the 2.2 replacement rate (the number of children needed to replace the parents and compensate for child mortality).

This represents an 18.2% decline from the 2.75 reported in 2000 and – according to a recent projection by the Institute for Health Metrics and Evaluation – the TFR may drop to 1.83 by 2050, with significant implications for population health and socio-economic sustainability.

“The decline is most pronounced in highly urbanized, higher-income countries such as Japan, South Korea and Singapore, the latter of which currently is reporting a TFR of 0.97, but it is making itself felt in developing countries too, especially those that are rapidly urbanizing,” says Nathalie Roebbel, cross-cutting lead of the World Health Organization’s (WHO) Urban Health unit in the Department of Social Determinants of Health.

Thailand is one example, the national average TFR standing at around 1.3 and 1.1 in urban areas. India is another, a country with a national average TFR of 2.1 and 1.6 in urban areas.

Poonam Muttreja, executive director of the Population Foundation of India, believes that only the first of those two numbers is the result of government policy.

“In 1952, India became the first country in the world to launch a national family planning programme,” she says. “Since then, successive governments have promoted smaller family size as an important development goal, but what is happening in urban areas and especially in cities is different: it is not the result of policy but of conditions arising in cities themselves.”

While acknowledging that not all cities are the same, Muttreja believes that certain conditions are sufficiently prevalent in most to be considered characteristic if not defining.

Among them is the relative cost of child bearing and rearing in urban as opposed to rural areas. In rural areas, children often contribute to the family as a source of free labour and income, in the cities they typically represent a financial burden.

“What is happening in the cities is different.”

Poonam Muttreja

Caring for children also becomes more challenging in urban environments. “In rural villages in India, the extended family and wider community often help with child-rearing,” Muttreja notes. “However, in cities, the family usually consists only of parents and their children, and both parents often work. And because the government has not invested in childcare, urban parents typically face the additional expense of hiring someone to care for their children.”

Similar problems are faced by urban parents across the country income spectrum, including those high-income countries implementing pro-natal policies. In Singapore, for example, where the government introduced a Baby Bonus Scheme in 2001 that includes a cash gift distributed over a child’s early years, parents still struggle.

“I have yet to meet a parent in any of our studies who feels that the measures significantly reduce the financial stress of having children,” says Kalpana Vignehsa, a senior research fellow at the Institute of Policy Studies in Singapore. “Indeed, because health care is paid out of pocket in Singapore, many parents say they have exhausted most of the Baby Bonus by the time they have left the hospital after giving birth.”

And the expenditure does not end there. “Caring for, educating and entertaining a child in a busy metropolis is expensive and many dual-income parents struggle to afford it.” Vignehsa explains.

Varaprasad concurs, pointing out nevertheless that competitive parenting is an additional factor. “The problem is childrearing has become something of an arms race,” she says, “with parents clamouring to give their children what they perceive to be the best of everything – the best schools, additional classes, etc, with all the expense and pressure that entails.”

Anna Rottkirch, a sociologist and demographer at the Population Research Institute of the Family Federation of Finland, who is currently producing a report for the government regarding fertility trends in a country where 8 in 10 people live in an urban area, believes that in many cases it is the ability to choose that is contributing to the falling TFR.

Like other Scandinavian countries, Finland has often been held up as exemplary in maternity care, parental leave, and childcare provision, achieving a relatively robust TFR and high levels of female workforce participation. However, like other Scandinavian countries, Finland has seen its TFR fall in recent years, dropping from 1.87 in 2010 to 1.37 in 2023.

“In some ways we are seeing the downside of our achievements,” says Rottkirch. “For example, the decrease in accidental pregnancies in the country is exactly what organizations such as mine have supported by advocating for access to free contraceptives for those under 25.”

However, access to contraceptives has transformed parenthood into a more deliberate choice. Rottkirch, herself mother of three children, argues that evolution has programmed humans to conceive and then look after the offspring, not to make conscious decisions about whether to have children. “When it becomes elective, it typically leads to postponement, with couples focusing on educational pursuits and career-building. This has significant implications for family size,” she says.

Muttreja observes the same phenomenon in India’s cities. “Young women want to further their education and find a job, and because they have good access to contraception, they delay having children and then it becomes harder,” she says.

Rottkirch believes that any response to the TFR challenge needs to be comprehensive, a view shared by Roebbel, who advocates for a multifaceted strategy that “takes full account of the social determinants impacting the TFR and goes beyond traditional health sector boundaries.”

Both women believe it is vital that pro-natal policies and initiatives already in place should be maintained if not reinforced. Vignehsa takes the same view, arguing for an extension of the Government of Singapore’s support package.

Roebbel also argues for a greater readiness to consider what are often controversial questions. “Historically, demographic debates have been contentious, often focusing on whether population growth impedes economic development or environmental sustainability, and there have been several instances of heavy-handed interventions to adjust the TFR,” she says.

According to *Demographic change and urban health: towards a novel agenda for delivering sustainable and healthy cities for all,* a piece of research commissioned by the WHO Urban Health unit and published on the F1000 research platform, the focus changed with the 1994 Cairo Programme of Action, with attention shifting to individual rights, particularly women's reproductive health and freedom from coercion. Since then, according to the authors, there has been a tendency to avoid discussing the topic in any other terms.

“We are seeing the downside of our achievements.”

Anna Rottkirch

Rottkirch agrees and, while supporting freedom of choice and acknowledging the wide diversity of interpersonal relationships, believes a start could be made by looking at the narrative around motherhood and partnerships.

“We need to be increasing fertility awareness through education on reproductive biology and the optimal timing for having children, supporting partnerships by emphasizing the benefits of committed relationships, while avoiding overtly pro-natalist policies of the kind being used in some authoritarian regimes,” she says.

Vignehsa also argues for a more balanced discussion: “It’s great that people are no longer pressured into having children, and that choosing not to have children doesn't affect one's status,” she says. “However, it's crucial to support those who do choose to become parents.”

All those interviewed for this article highlighted the importance of equity in gender roles in heterosexual couples. “In Singapore, women are still expected to be largely responsible for childcare and home care even they share responsibilities in the workplace,” says Vignehsa. “This is neither equitable nor sustainable.”

This imbalance was one of the things that gave Varaprasad pause when she was considering motherhood. “Social norms put pressure on the woman,” she says. “I would say my husband is pretty good about it all, but I still do most of the childrearing, despite working as hard as he does. I think a lot of my friends hesitate because they don’t want to have to deal with that.”

**Figure:**
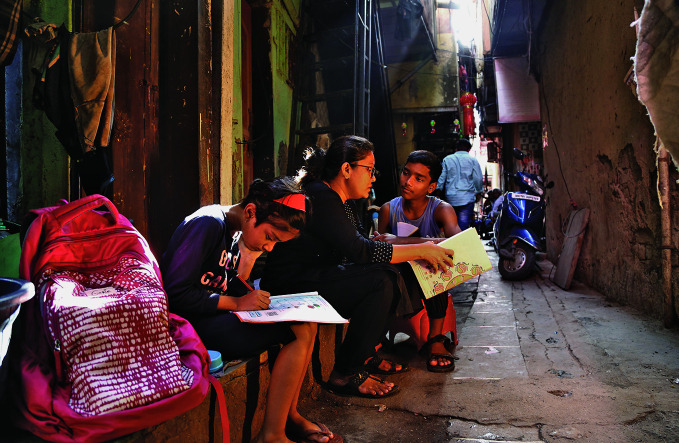
A mother teaching her children in Matunga, Mumbai, India.

UNICEF/ Faisal Magray

**Figure:**
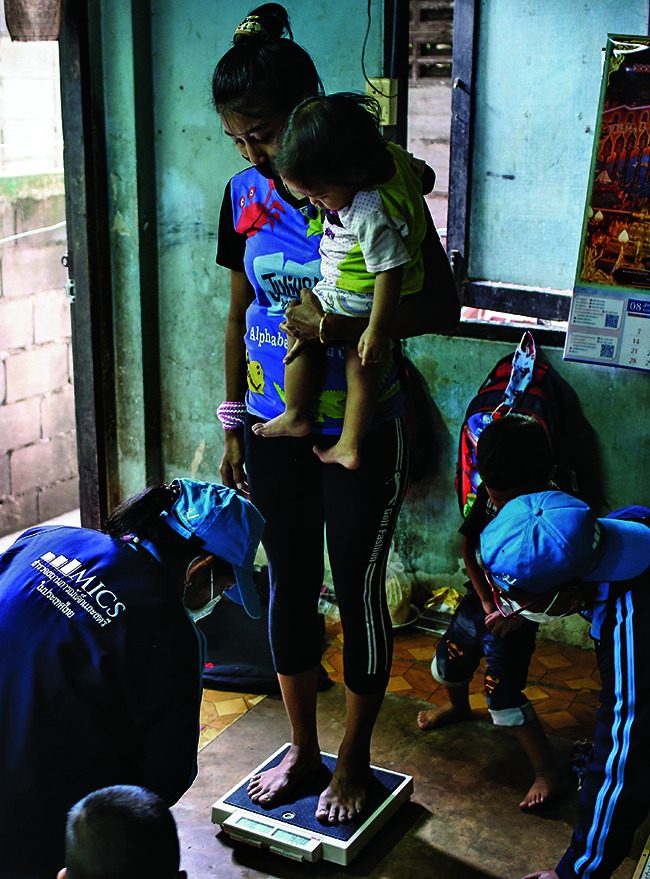
Recording the weight of a mother and child in Bang Sue, Bangkok, Thailand.

UNICEF/ Sukhum Preechapanich

